# Open Surgery including Lymphadenectomy without Adjuvant Therapy for Uterine-Confined Intermediate- and High-Risk Endometrioid Endometrial Carcinoma

**DOI:** 10.3390/curroncol29050298

**Published:** 2022-05-19

**Authors:** Isao Otsuka, Takuto Matsuura, Takahiro Mitani, Koji Otsuka, Yoshihisa Kanamoto

**Affiliations:** Department of Obstetrics and Gynecology, Kameda Medical Center, Kamogawa 296-8602, Chiba, Japan; matsuura.takuto@kameda.jp (T.M.); mitani.takahiro@kameda.jp (T.M.); otsuka.koji@kameda.jp (K.O.); kanamoto.yoshihisa@kameda.jp (Y.K.)

**Keywords:** endometrioid endometrial carcinoma, open surgery, lymphadenectomy, intermediate-risk, high-risk, adjuvant therapy, recurrence

## Abstract

Minimally invasive surgery may not be an appropriate surgical approach in intermediate- and high-risk endometrial carcinoma, even though adjuvant therapy is given. The objective of this study was to evaluate the results of open surgery including lymphadenectomy without adjuvant therapy in patients with uterine-confined intermediate- and high-risk endometrioid endometrial carcinoma. Two hundred fifty-six patients with uterine-confined endometrioid endometrial carcinoma were treated with open surgery, including pelvic with or without para-aortic lymphadenectomy. Of the 81 patients with uterine-confined intermediate- or high-risk disease, 77 were treated with systematic lymphadenectomy without adjuvant therapy. Seven patients developed recurrence, comprising 5.5% (3/55) and 18.2% (4/22) of the intermediate- and high-risk patients, respectively. The time to recurrence was 1–66 months. The sites of recurrence were the vaginal apex (*n* = 2), lung (*n* = 2), vaginal sidewall (*n* = 1), pelvic lymph nodes (*n* = 1), and para-aortic to supraclavicular nodes (*n* = 1). Of these, five patients were alive without disease after salvage treatment, but two understaged high-risk patients died of disease. The five-year disease-specific survival rates of intermediate- and high-risk patients were 100% and 90%, respectively. The present study indicated that patients with uterine-confined intermediate- and high-risk endometrioid endometrial carcinoma had excellent survival when treated with open surgery, including lymphadenectomy alone. The safety of omitting adjuvant therapy should be evaluated in prospective randomized trials comparing open surgery with minimally invasive surgery.

## 1. Introduction

Endometrial cancer is the most common gynecological tract cancer in developed countries. Endometrial cancer can be classified as low-, intermediate-, and high-risk disease based on the pathological features. The cornerstone of treatment is surgery, and recently, minimally invasive surgery, i.e., laparoscopic and robotically-assisted surgery, is considered to be the standard surgical approach based on the results of two randomized studies [1,2]. In addition, pelvic lymphadenectomy may be omitted, as two randomized studies did not show its therapeutic effects [3,4].

However, the results of these studies may not apply to intermediate- and high-risk endometrial cancer because these studies were performed on patients of all risk groups, most of whom have low-risk disease. In addition, patients in these studies could receive post-operative therapy that may obscure the effects of surgery itself. Most recently, many studies show that intermediate- and high-risk patients who underwent minimally invasive surgery are at higher risk of recurrence than those who underwent open surgery [5,6,7,8,9,10].

Moreover, in intermediate- and high-risk diseases, lymphadenectomy appears to have both diagnostic and therapeutic effects [11]. Previous studies reported that open surgery, including lymphadenectomy, was associated with excellent survival rates in patients with surgically-confirmed uterine-confined diseases even though they do not receive adjuvant therapy [12,13,14]. Lymph node recurrence rarely developed after systematic lymphadenectomy in endometrioid endometrial carcinoma [15,16,17]. Considering the results of these studies, in our institution, adjuvant therapy has not been administered in patients with uterine-confined endometrioid endometrial carcinoma who underwent open surgical staging, including lymphadenectomy, since 2004. The objectives of this study were to evaluate the results of open surgery including lymphadenectomy without adjuvant therapy in patients with uterine-confined intermediate- and high-risk endometrioid endometrial carcinoma, and to evaluate whether adjuvant therapy can be safely omitted without decreasing long-term survival.

## 2. Patients and Methods

The patients included in the present study were those with intermediate- and high-risk endometrioid endometrial carcinoma who underwent surgical staging including lymphadenectomy and had no extrauterine disease, treated at our institution between 2004 and 2020. The risk groups were defined according to the definitions provided by the Japan Society of Gynecologic Oncology: low-risk (grade 1 or 2 tumor with <1/2 myometrial invasion), intermediate-risk (grade 1 or 2 tumor with ≥1/2 myometrial invasion, or grade 3 tumor with <1/2 myometrial invasion, and lymphovascular space invasion), and high-risk (grade 3 tumor with ≥1/2 myometrial invasion, and cervical stromal invasion) [18]. The tumors of all patients were staged according to the 2009 International Federation of Gynecology and Obstetrics (FIGO) classifications. The tumor subtype was assessed on the basis of morphological assessment of hematoxylin and eosin-stained slides by our institutional pathologists, with the use of immunohistochemistry as a diagnostic adjunct when necessary. Patients with high-risk histology (serous carcinoma, clear cell carcinoma, mixed carcinoma, and carcinosarcoma) and patients with synchronous ovarian carcinoma were excluded.

Our practice for patients with endometrial carcinoma is as follows: surgical staging consisting of total abdominal hysterectomy, bilateral salpingo-oophorectomy, pelvic lymphadenectomy, and cytologic testing of peritoneal washing was performed. The para-aortic nodes up to the renal vessels were dissected in patients at risk of para-aortic node metastasis, i.e., those with deep myometrial invasion determined intraoperatively by gross inspection of a sectioned uterine corpus, grossly positive pelvic or para-aortic nodes, or gross adnexal metastasis [15,19]. Lymphadenectomy was performed through open surgery, as open surgery is necessary to completely dissect the pelvic and para-aortic lymph nodes. Pelvic and para-aortic lymphadenectomy procedures have been described previously [15]. Left para-aortic lymphadenectomy was performed and the sympathetic nerves over the descending aorta were preserved. Lymphadenectomy was omitted in elderly patients with comorbidities and morbidly obese patients. Laparoscopic surgery that was introduced relatively recently has been performed only in patients with small, low-grade endometrioid tumors with superficial myometrial invasion. In principle, no adjuvant therapy was administered to surgically staged patients without extrauterine diseases, which includes positive peritoneal cytology.

The disease-free survival time was calculated from the date of surgery to the date of recurrence or last contact. The disease-specific survival time was calculated from the date of surgery to the date of death or last contact. The survival times of patients who died of causes other than endometrial carcinoma were censored at the date of death. The disease-free and disease-specific survival rates were estimated using the Kaplan-Meier method. This study was approved by the institutional review board.

## 3. Results

### 3.1. Surgical Staging

During the study period, 478 patients with endometrial carcinoma underwent surgical treatment, and 363 of these patients had endometrioid endometrial carcinoma without synchronous ovarian cancer. Of these, 292 patients underwent open surgical staging, including lymphadenectomy. All of the patients underwent pelvic lymphadenectomy, and 41 patients underwent para-aortic lymphadenectomy. The mean and median numbers of pelvic and para-aortic lymph nodes removed were 19.4 and 19 (range: 1–73) and 8.3 and 8 (1–21), respectively. Lymph node metastasis was observed in 27 (9.2%) patients; pelvic and para-aortic node metastases were observed in 22 (7.5%) and 8 (2.7%) patients, respectively (Table 1). Three (1.0%) patients had isolated para-aortic lymph node metastasis without pelvic node involvement. The distribution of FIGO stages was as follows: stage IA, 193 patients (66.0%); IB, 49 (16.7%); II, 15 (5.1%); IIIA, 5 (1.7%); IIIB, 2 (0.7%); IIIC1, 19 (6.5%); IIIC2, 8 (2.7%); and IVB, 1 (0.3%).

### 3.2. Patient Demographics

Of the 257 patients with stage I/II endometrioid endometrial carcinoma, one patient had positive peritoneal cytology. Of the 256 patients without extrauterine disease, 175 (68.3%), 56 (21.9%), and 25 (9.8%) patients had low-, intermediate-, and high-risk diseases, respectively. Of the 81 intermediate- and high-risk patients, three high-risk patients who requested and received adjuvant chemotherapy and one elderly (76 years) intermediate-risk patient who underwent lymphadenectomy but only one node removed were excluded. Thus, 77 patients, consisting of 55 intermediate-risk and 22 high-risk patients, were treated with open surgery including lymphadenectomy alone. The median age was 62 years (range: 34–80 years). The median follow-up period was 75 months (range: 7–203 months). Only six patients (7.8%) were obese (body mass index ≥30 kg/m^2^).

### 3.3. Patterns of Recurrence and Long-Term Survivals

Of the 77 patients, seven developed a recurrence (Table 2). Of these, four patients were salvaged with radiotherapy: two with vaginal, one with pelvic node, and one with solitary pulmonary recurrence. Another patient who developed pulmonary recurrence was alive without disease after salvage treatment consisting of chemotherapy and surgery.

Two other patients, who had been identified as high-risk cases, died of the recurrent disease. One of these two patients underwent pelvic lymphadenectomy alone because of medical comorbidities, although deep myometrial invasion (>2/3) was revealed by intraoperative gross examination of the sectioned uterine corpus. She refused to undergo computed tomography (CT) scans during follow-up and subsequently noticed a left supraclavicular node swelling 28 months after surgery. Lymph node swelling in the para-aortic, mediastinal, and supraclavicular regions was detected using positron emission tomography/computed tomography (PET/CT). She refused salvage therapy and received the best supportive care. The other patient who died had a stage IB grade 3 tumor that protruded through the cervical os and adhered densely to the vaginal sidewall, where a recurrent tumor developed one month after the surgery, indicating that the patient actually had extrauterine disease (vaginal metastasis) at surgical staging. She received salvage radiotherapy but subsequently developed inguinal node metastasis on the same side of the vaginal sidewall.

**Figure 1:**
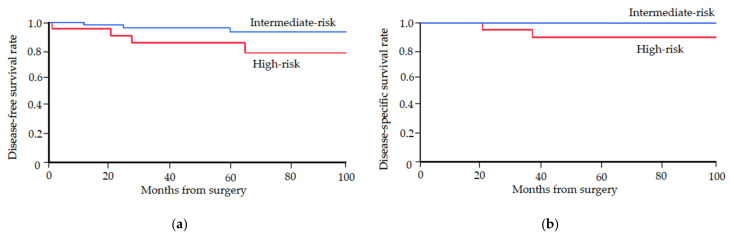
Kaplan-Meier survival curves for uterine-confined intermediate- and high-risk groups. (**a**) Disease-free survival. (**b**) Disease-specific survival.

The five-year disease-free survival rates of intermediate-risk and high-risk patients were 96% and 85%, respectively (Figure 1a). The five-year disease-specific survival rates of intermediate-risk and high-risk patients were 100% and 90%, respectively (Figure 1b). The five-year disease-free and disease-specific survival rates of intermediate/high-risk patients were 93% and 97%, respectively.

## 4. Discussion

The present study indicates that patients with uterine-confined intermediate- and high-risk endometrioid endometrial carcinoma had excellent survival when treated with open surgery, including lymphadenectomy alone.

Compared with open surgery, minimally invasive surgery has lower surgical morbidity and the need for shorter hospital stays [20]. However, to date, no firm evidence that the effect of minimally invasive surgery in intermediate- and high-risk patients on long-term survival is equivalent to that of open surgery has been established. A randomized trial where lymphadenectomy was performed in all patients failed to show the non-inferiority of laparoscopy [1]. Another randomized trial showed that the use of open hysterectomy and laparoscopic hysterectomy resulted in equivalent survival outcomes [2]. However, in that trial, only selected surgeons performed surgery on a highly selected group of endometrioid carcinoma patients. Notably, non-obese women tended to have a better long-term DFS when treated with open surgery (86.6% vs. 77.4%, *p* = 0.060) [2]. Non-obese women, consisting of 92.2% of the patients in the present study, are known to have a higher risk of metastatic disease compared to obese women, as non-obesity is associated with more aggressive disease and worse prognosis [21,22].

Most recently, minimally invasive surgery has been reported to be related to higher recurrence rates compared to open surgery in intermediate- and high-risk patients [6,8]. Robotic surgery was also associated with a higher recurrence rate in patients with intermediate-risk endometrioid carcinoma even though they received adjuvant radiation [5]. In patients with high-grade tumors, extracting a large uterus increased the risk for intra-abdominal recurrence significantly [7]. Although studies on the use of a uterine manipulator on oncological outcomes have reported mixed results, its use might be associated with a higher risk of death in patients treated with minimally invasive surgery [23,24]. These results suggest that tumor spillage may be a mechanism for recurrence [7,24], similar to patients with cervical cancer treated with minimally invasive surgery. In addition, certain molecular features of endometrial cancer may be correlated with worse survival outcomes when treated with minimally invasive surgery. Minimally invasive surgery accelerated the recurrence of microsatellite-stable endometrioid cancer [9] and TP53-mutated cancer [10]. Although overall survival rates were not decreased when treated with minimally invasive surgery, salvage treatment for recurrent diseases may decrease the long-term quality of life and increase the total cost of treatment.

Systematic lymphadenectomy appears necessary in patients with intermediate- and high-risk diseases, as they are at a higher risk of lymph node metastasis compared to patients with low-risk disease. In endometrioid carcinoma, lymphadenectomy that removes both macroscopic and microscopic nodal metastases appears to improve survival [25,26]. Additionally, lymphadenectomy can remove lymph node metastases that are only detectable by ultrastaging using serial sections and immunohistochemical staining. In the present study, only one lymph node recurrence, which was diagnosed radiologically, developed. This suggests that systematic lymphadenectomy effectively removes microscopic metastases that are undetectable by conventional histology. Moreover, para-aortic lymphadenectomy appears to be necessary for intermediate- and high-risk patients since para-aortic node metastasis is almost exclusively observed in these patients [15,19]. In contrast to pelvic lymphadenectomy alone that had no significant effect on improving survival in intermediate- and high-risk patients, para-aortic and pelvic lymphadenectomy has been shown to improve survival [11,27,28]. Recently, we reported that low-volume para-aortic node metastasis could be cured by surgery including lymphadenectomy alone [29].

The omission of adjuvant therapy may not be safe in patients with negative sentinel nodes, whereas sentinel node biopsy leads to the omission of systematic lymphadenectomy. In early-stage endometrial carcinoma, ultrastaging that may be difficult to perform during surgery is necessary to detect low-volume metastases, which comprise approximately half of lymph node metastases [30,31]. In node-negative patients, those who underwent sentinel node biopsy were less likely to omit adjuvant therapy than those who underwent systematic lymphadenectomy [32], and nodal recurrence developed in some patients with negative sentinel nodes [33].

Adjuvant therapy does not appear necessary in patients with uterine-confined intermediate- and high-risk endometrioid carcinomas who underwent open surgical staging, including lymphadenectomy. Previous studies have reported excellent long-term survival rates of patients treated with open surgery including lymphadenectomy alone (Table 3) [12,13,14]. In the present study, only two understaged patients with high-risk disease died of their disease: One patient who might have occult para-aortic lymph node metastasis and the other patient whose vaginal involvement was missed. After the exclusion of these patients, none of the five patients who developed recurrence died of disease during the study period. Vaginal radiotherapy, which is more likely to be given after minimally invasive surgery compared to open surgery [20] and may reduce the quality of life, can be omitted after open surgery, as the majority of isolated vaginal recurrences in non-irradiated patients can be cured with salvage radiotherapy [34]. Adjuvant external beam radiotherapy to the pelvis does not improve survival, although it decreases pelvic recurrence [35,36]. Platinum-based chemotherapy is more effective than radiotherapy in intermediate- and high-risk patients [37,38]. However, chemotherapy does not appear necessary in uterine-confined endometrioid carcinoma, as hematogenous metastasis is rare in these patients. In the present study, only two patients developed pulmonary metastasis, which occurred more than five years after surgery. Notably, of the three patients with high-risk disease who were excluded from this study because of the receipt of paclitaxel/carboplatin chemotherapy, one patient with a grade 3 tumor developed pulmonary recurrence. Current chemotherapy may not be effective in grade 3 tumors [39,40].

The risk classification based on the presence of extrauterine disease may be simpler than that based on uterine pathological factors. Uterine pathological risk factors, i.e., deep myometrial invasion, high-grade tumor, lymphovascular space invasion, and cervical involvement, are identical to the risk factors for lymph node metastasis [41]. The prognostic value of these factors may disappear when lymphadenectomy is performed, and no lymph node metastasis is detected.

The limitations of this study include the small number of patients, as this is a single-institution study, and intermediate- and high-risk tumors were only 31.7% of uterine-confined endometrioid tumors. Additionally, the number of lymph nodes removed varied among patients. However, the nodal count may not always be indicative of the extent of the lymph-node dissection. The systematic removal of all lymphatic tissue might be the most accurate definition of a complete lymphadenectomy [27]. Lastly, molecular analysis that is useful to tailor adjuvant treatment [42,43] was not performed. Molecular alterations, such as mismatch repair protein deficiency and p53 abnormalities, may be more effective in predicting the risk of extrauterine diseases than uterine pathological factors [44,45]. However, these molecular alterations may be associated with extrauterine disease, similar to uterine pathological risk factors, but not be predictive for survival in surgically-staged patients with uterine-confined endometrioid carcinoma.

The strengths of this study include its simple design. The surgical procedures had not been changed during the study period and the heterogeneity of surgical management that might compromise the effects of complicated surgery in multi-center studies was avoided. The exclusion of high-risk histology is another strength. High-risk histological subtypes, such as serous carcinoma, appear to have a distinct disease spread pattern that cannot be eradicated by surgery alone.

## 5. Conclusions

In conclusion, patients with intermediate- and high-risk endometrioid endometrial carcinoma who are treated with open surgery, including lymphadenectomy, may not need adjuvant therapy when extrauterine diseases are not detected. Systematic lymphadenectomy should be performed in patients with apparent intermediate- and high-risk endometrial carcinoma since adjuvant therapy may not eradicate the residual disease in the lymph nodes. The performance of lymphadenectomy seems justified, as it does not affect the long-term quality of life [46]. The safety of omission of adjuvant therapy after systematic lymphadenectomy in these patients needs to be evaluated in prospective randomized trials. Specifically, randomized trials comparing open surgery with minimally invasive surgery are necessary for intermediate- and high-risk endometrioid carcinoma. In the future, precision treatment consisting of adequate surgery and adjuvant therapy based on precise pre-, intra-, and post-operative evaluation using imaging, immunohistochemical, and molecular studies will be necessary to avoid both under- and over-treatment.

## Figures and Tables

**Table 1:** Incidence of lymph node metastasis by various surgical-pathological factors.

	No. of Patients	Lymph Node Metastasis			
	(*n* = 292)	Total (*n* = 27)	Pelvic (*n* = 24)	Para-Aortic (*n* = 8)	Para-Aortic Alone (*n* = 3)
**pT classification**					
pT1A	200	7 (3.5%)	7 (3.5%)	0	0
pT1B	62	12 (19.4%)	11 (17.7%)	3 (4.8%)	1 (1.6%)
pT2	17	2 (11.8%)	2 (11.8%)	0	0
pT3	13	6 (46.2%)	4 (30.8%)	5 (38.5%)	2 (15.4%)
**Tumor Grade**					
G1	155	8 (5.2%)	7 (4.5%)	1 (0.6%)	1 (0.6%)
G2	94	10 (10.6%)	10 (10.6%)	4 (4.3%)	0
G3	43	9 (20.9%)	7 (16.3%)	3 (7.0%)	2 (4.7%)
**Myometrial invasion**					
<1/2	214	9 (4.2%)	9 (4.2%)	1 (0.5%)	0
≥1/2	78	18 (23.1%)	15 (19.2%)	7 (9.0%)	3 (3.8%)
**Cervical stromal invasion**					
No	268	22 (8.2%)	20 (7.5%)	5 (1.9%)	2 (0.7%)
Yes	24	5 (20.8%)	4 (16.7%)	3 (12.5%)	1 (4.2%)
**Lymphovascular space invasion**					
Negative/Undetermined	246	11 (4.5%)	9 (3.7%)	3 (1.2%)	2 (0.8%)
Positive	46	16 (34.8%)	15 (32.6%)	5 (10.9%)	1 (2.2%)
**Risk group †**					
Low-risk	175	5 (2.9%)	5 (2.9%)	0	0
Intermediate-risk	72	11 (15.3%)	10 (13.9%)	3 (4.2%)	1 (1.4%)
High-risk	45	11 (24.4%)	9 (20.0%)	5 (11.1%)	2 (4.4%)

† Risk group was classified by uterine factors alone.

**Table 2:** Characteristics of the patients with uterine-confined endometrioid endometrial carcinoma who developed recurrence.

Case	Age	Risk	G, MI, Cx, LVSI	LA	Site of Rec	Time to Rec	Salvage Treatment	Status	Survival after Rec
1	58	Intermediate	G1, ≥1/2, No, No	P	Lung	61 mo	Chemo, Surgery	NED	37+ mo
2	70	Intermediate	G1, ≥1/2, No, No	P	Vaginal apex	25 mo	RT (EBRT + VB)	NED	81+ mo
3	64	Intermediate	G2, ≥1/2, No, Yes	P + PA	Pelvic LN †	12 mo	RT (EBRT)	NED	125+ mo
4	67	High	G3, ≤1/2, Yes, Yes	P	Vaginal apex	21 mo	RT (EBRT + VB)	NED	58+ mo
5	57	High	G3, ≥1/2, No, Yes	P	Vaginal sidewall	1 mo	RT (EBRT + VB), Chemo	DOD	20 mo
6	71	High	G3, ≥1/2, No, Yes	P + PA	Lung	66 mo	RT	NED	11+ mo
7	70	High	G2, ≥1/2, Yes, Yes	P	Para-aortic—supraclavicular LN	28 mo	None	DOD	10 mo

G, grade; MI, myometrial invasion; Cx, Cervical stromal invasion; LVSI, lymphovascular space invasion; LA, lymphadenectomy; Rec, recurrence; P, pelvic; PA, para-aortic; † LN, lymph node; RT, radiation therapy; EBRT, external beam radiotherapy; VB, vaginal brachytherapy; NED. No evidence of disease; DOD, dead of disease; †diagnosed radiologically.

**Table 3:** Five-year survival rates of patients with uterine-confined intermediate- and high-risk endometrial carcinoma treated with open surgery alone.

Authors (Year)	Cases (No.)	Stage, Grade; Histology	Lymphadenectomy (LA)	5-Year Survival Rate	Median Follow-Up
Chen (1989)	18	IAG3, IB	Selective biopsy of pelvic and para-aortic lymph nodes	100% (DFS)	5–13 years
Ayhan (2002)	25	IAG3, IB; endometrioid	Pelvic and para-aortic LA	92% (OS)	96 months
Straughn (2003)	121	IB; serous and clear cell were excluded	Pelvic and para-aortic LA	90% (OS)	41 months
Present study	77	IAG3, IB, II; endometrioid	Pelvic LA in all patients and para-aortic LA in selected patients	97% (DSS)	75 months

DFS, disease-free survival; OS, overall survival; DSS, disease-specific survival.

## Data Availability

The data presented in this study are available on request from the corresponding author.
